# Early experience on injectable micronized putty type human‐derived acellular dermal matrix (ADM) in management of diabetic foot wounds in Singapore

**DOI:** 10.1111/iwj.70127

**Published:** 2025-01-12

**Authors:** Alison Mei Fern Quah, Marcus Jia Ming Ng, Li Zhang, Yam Meng Chan, Shufen Neo, Malcolm Mak, Qiantai Hong, Glenn Tan, Ying Pan, Enming Yong

**Affiliations:** ^1^ Vascular Surgery Unit, Department of General Surgery Tan Tock Seng Hospital Singapore Singapore; ^2^ Skin Research Institute of Singapore, Agency for Science Technology and Research (A*STAR) Singapore Singapore

**Keywords:** acelluar dermal matrix, diabetic foot wounds, lower limb wounds, paste, putty matrix, wound healing

## Abstract

Diabetic foot wounds (DFW) are notoriously difficult to treat owing to poor vascularity, delayed healing and higher rates of infection. Human‐derived acellular dermal matrices (ADM) have been used in DFW treatment, utilizing a matrix scaffold for new tissue generation. We investigate the efficacy of a micronized injectable human‐derived ADM in the treatment of DFW. We retrospectively recruited 13 patients with diabetic foot wounds. Wounds were adequately debrided, and a micronized injectable ADM was applied. Wound sizes were recorded prior to treatment, at 2 and 4 weeks post‐treatment. The mean defect of wounds treated was 19.21 cm^3^. Our results showed a statistically significant reduction in wound size of 45% and 59% at 2 and 4 weeks post‐treatment, respectively (*p* < 0.01). ADM was also effective in infected DFW as 84% of our wounds had positive tissue cultures at the time of application. Micronized injectable ADM has proven to be an effective treatment for DFW. Advantages include a ready‐to‐use injectable, single‐stage treatment, minimal pain, mouldable matrix to fit any wound shape, allows for outpatient treatment and simple wound dressings.

## 1 INTRODUCTION

Diabetic foot wounds (DFW) are challenging and complex. Underlying pathophysiological factors, such as peripheral neuropathy, impaired vascularity and angiogenesis, impaired immunological response all result in increased susceptibility to infection.^1^ These factors often contribute to the difficulty in achieving successful wound healing outcomes often leading to lower limb amputations. Patients with diabetic foot ulcers face a higher mortality risk, with a 5‐year mortality rate of 37.9% for those requiring major amputations.^2^

Management of DFWs requires a multidisciplinary team that can address the various components of this condition, including debridement of necrotic tissue, administration of appropriate antibiotic therapy, achieving timely wound closure and implementation of offloading strategies to reduce the incidence of recurrence.

Acellular dermal matrix (ADM) has been employed as an adjunct for wound healing since 1994.^3^ ADMs can be manufactured from allogenic or xenogeneic sources by removing cellular components while leaving and extracellular scaffold and growth factors that signal for cellular migration, proliferation and angiogenesis. Eventually, these matrices will be repopulated and remodelled by the patient's fibroblast and endothelial cells.^4^, ^5^, ^6^

Most ADMs are manufactured as sheets which is useful in resurfacing large wounds. However, diabetic foot wounds and post‐surgical diabetic wounds may be smaller and deep, making the application of sheet ADMs challenging. The size and location of the wounds may also preclude application of advanced wound dressings like negative pressure wound therapy as the seal may be difficult to obtain between the toes. Micronized ADMs in the form of a paste (putty) enable easy application, allowing the putty to fill deep cavities and conform to irregular surfaces.

This study retrospectively examines the efficacy of our institution's early experience with the use of micronized human‐derived ADMs in putty form for management of DFWs.

## 2 MATERIALS AND METHODS

### 2.1 Study design and population

This retrospective study was conducted at Tan Tock Seng Hospital, which is a 1800‐bed tertiary hospital in Singapore. The study cohort consisted of 13 patients with 18 DFWs. Post‐surgically debrided or minor amputation wounds were also included. Post‐ray amputation wounds that were not suitable for primary or delayed primary closure were considered as the study team postulated that these wounds would benefit due to the location and depth of such wounds making application of negative pressure wound therapy challenging. We did notrequire them to have negative tissue cultures prior to treatment. Still, the patients must not display signs of clinical infection, and culture‐directed antibiotics were continued as per infectious disease advice. Patients with evidence of significant lower limb ischemia on arterial duplex underwent necessary revascularization (angioplasty or bypass). Patients with uncontrolled infection requiring further surgical debridement, uncontrolled autoimmune disease, active chemotherapy or radiotherapy were excluded. Ethics approval for the study was obtained from our hospital's Institutional Review Board (NHG DSRB Reference: 2023/00544).

### 2.2 About micronized injectable human‐derived ADMs

The ADM applied consists of a putty/paste type wound dressing (CG Reallo Putty™, CG Bio, Seongnam‐si, Gyeong‐gi‐do, South Korea) derived from micronized human acellular dermal matrix and sterile distilled water with an average particle size of 650 μm.^7^ The matrix is an allograft obtained from human donor skin tissue banks. The skin is de‐epithelized and de‐cellularized before creating a sheet‐type ADM which is then micronized into powder form before being prepared into a paste (putty) form.

Its mechanism of action, like many ADMs, includes promoting granulation and wound healing by providing an extracellular scaffold to the wound to support cellular ingrowth, facilitation of cell migration, proliferation and differentiation by initiation of signalling molecules, release of growth factors and stimulation of angiogenesis through activation of endothelial cells. The particular advantage of this paste (putty) formulation includes the ease of use on irregular and deep wound beds by allowing for better ADM contact.

### 2.3 Intervention and measurement

All patients underwent adequate surgical debridements of their DFW or surgical amputation of the toe(s) as needed and were started on culture‐directed antibiotics. Patients with evidence of significant arterial occlusion underwent revascularization to ensure optimal blood supply. There were 13 patients with a total of 18 DFWs/post‐surgical wounds that were evaluated. After the wound bed was assessed to be clean, micronized injectable human‐derived putty ADM was administered directly into the wound bed to fill the wound in entirety. Putty ADM was gently packed within the wound to prevent dead space, and the wound was dressed with a non‐adhesive foam dressing. The application of the putty ADM was once‐off. Secondary foam dressings were changed twice weekly to manage the wound exudate. The matrix typically displayed full absorption by Day 7. Following which, the wound would be dressed with conventional dressings with foam dressing. Hydrofibre (Aquacel Ag™, Convatec, London, United Kingdom) was placed under the foam dressing into the wound bed cavity, if the wound bed cavity was still present.

Wound dimensions were recorded manually using a ruler. Measurements of the length, width and depth of each wound were taken, and the volume was calculated at baseline (Day 0), and on Days 14 and 28. Absolute reduction of wound volume was used to document the rate of wound healing. Time to wound healing and the need for subsequent major amputation and mortality were also recorded.

### 2.4 Statistical analysis

Statistical analysis was conducted using GraphPad Prism version 8.0.2 for Windows (GraphPad Software, SAN Diego, California, USA). Student's *t*‐test was used on continuous data to compare the means between two groups. A *p*‐value of <0.05 was considered statistically significant. When the data set follows a normal distribution, the mean and standard deviation (SD) are reported. However, if the data do not follow a normal distribution, the median and interquartile range (IQR) are used instead.

## 3 RESULTS

### 3.1 Patient demographics and baseline characteristics

The cohort consisted of 13 patients, 8 males and 5 females, with a median age of 69 with IQR of 17 years. A total of 18 lower limb wounds were assessed as some patients had more than one wound. 10 (76%) patients had hypertension, and 4 (30%) patients had chronic kidney disease and 1 patient was on dialysis. 5 (38%) patients had previous DFWs. 5 (38%) patients were smokers, 9 (69%) patients required revascularization procedures. 11 (84%) patients had positive wound cultures at time of application but no signs of overt local or systemic infection. The patient demographics and baseline characteristics are as shown in Tables 1 and 2.

**TABLE 1:** Patient demographics.

Characteristics	*N* (percentage)
Study population	13 (100%)
Male: Female	8 (61%): 5 (38%)
Median age and interquartile range, years (IQR)	70 (17)
Median Hba1c (IQR)	7.1% (3.0)
HTN	10 (76%)
HLD	10 (76%)
CKD	4 (30%)
ESRF	1 (7%)
Smoker	5 (38%)
Revascularization	9 (69%)

Abbreviations: CKD, chronic kidney disease; ESRF, end‐stage renal failure; HLD, hyperlipidaemia; HTN, hypertension; *N*, number.

**TABLE 2:** Patient past medical history and WIFI score.

Patient	Age	Gender	Smoker	WIFI	Comorbidities	HbA1c	Revascularization
1	59	Male	N	3	HTN, DM	9.9	N
2	57	Female	N	4	ESRF, IHD, DM, HTN, HLD, PAD	6.2	Y
3	56	Female	N	4	HTN HLD DM PAD CCF CKD	7.1	Y
4	54	Male	Y	3	DM HLD	6.9	N
5	62	Male	N	4	DM IHD PAD	7.8	Y
6	84	Female	N	3	DM HTN HLD CKD PAD	6.5	Y
7	71	Male	N	4	DM HTN HLD CKD IHD PAD	7	Y
8	76	Male	Y	3	DM HTN HLD IHD	7	Y
9	73	Male	Y	3	DM CVI Heart block PAD, Anaemia, Stasis Eczema	6.4	Y
10	70	Male	Y	4	HTN HLD DM	17.4	Y
11	69	Male	Y	4	DM HTN HLD CVI Pancreatitis PAD	10.3	N
12	90	Female	N	4	HTN HLD DM AF CKD CHF Alzheimer's Dementia, Colorectal Cancer Gout	10.1	Y
13	76	Female	N	4	DM HTN HLD CVA	7.3	N

Abbreviations: AF, atrial fibrillation; CCF, congestive cardiac failure; CVI, chronic venous insufficiency; DM, diabetes melitus; IHD, ischemic heart disease; PAD, peripheral arterial disease; Y, yes; N, no.

### 3.2 Wound volume reduction

Significant reductions in wound volume were observed across the evaluated wounds. On Day 14, the mean wound volume decreased from 19.2 to 8.82 cm^3^ (*p* = 0.0026), indicating a mean reduction of 46.5% from baseline. Further reduction was noted by Day 28, with a mean wound volume of 5.8 cm^3^, corresponding to a total reduction of 62.5% (*p* = 0.0023) from baseline (Table 3).

**TABLE 3:** Wound characteristics and subsequent time to wound healing.

No	Location of wound	Pathology	Initial wound size (cm^2^)	Wound size at Day 14 (cm^2^)	Wound at Day 28 (cm^2^)	Time to wound healing (Weeks)	Further procedures/mortality/morbidity
1	Left third toe wound	Ischaemic	73.5	37.5	30	18	
2	Left lateral foot wound	Neuroischaemic	70	24.8	10.8	16	Deceased 1 year after admission secondary to ischemic heart disease
3	Left second toe ray amputation	Neuroischaemic	6.3	4.4	6	16	
4	Left transmetatarsal amputation	Neuroischaemic	16.8	6	1.8	12	
5	Left third to fifth toe amputation	Neuroischaemic	8	6.4	6.4	12	
6	Left second toe ray amputation	Ischaemic	37.8	29.2	20.3	18	Wound healed but readmitted for third toe infection requiring third toe ray amputation
7	Right third toe ray amputation	Ischaemic	7.5	4.5	5.6	23	
8	Left second toe ray amputation	Ischaemic	6.75	3	1.87	11	
9	Right big toe ray amputation	Ischaemic	18.4	5.75	2.5	Not healed	Deceased 6 months after application of ADM putty secondary to ischaemic heart disease
10	Left second toe ray amputation	Neuroischaemic	27	6	4.5	20	
11	Left lateral foot	Mixed arteriovenous	18	12	7.5	9	Split‐thickness skin graft applied 14 days after application of ADM putty
12	Left foot dorsum	Mixed arteriovenous	3.2	3.2	2.8	9	
13	Right foot dorsum	Mixed arteriovenous	0.6	0.3	0.3	9	
14	Right lateral malleolus	Mixed arteriovenous	4	4	0.4	9	
15	Left second toe ray amputation	Ischaemic	18	3	1.2	21	
16	Left third to fifth ray amputation	Ischaemic	6	0.8	0.38	16	
17	Left second toe amputation	Ischaemic	18	6	0.6	63	
18	Right second toe amputation	Ischemic	6	2	2	18	

**FIGURE 1:**
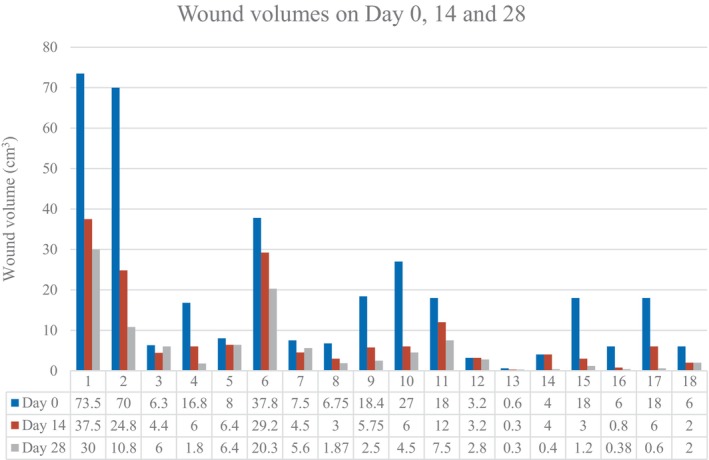
Patients' wound volume over Days 0, 14 and 28.

**FIGURE 2:**
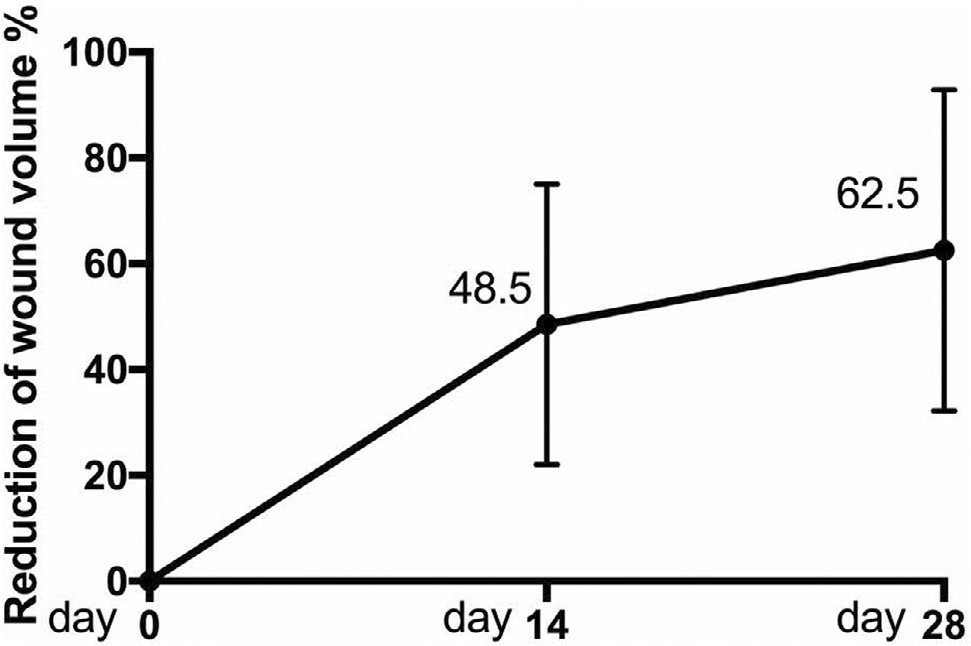
Mean percentage reduction in wound volume over 28 days. Error bars indicate standard deviation.

**FIGURE 3:**
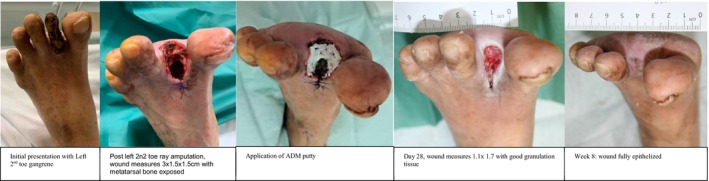
Case 1.

**FIGURE 4:**
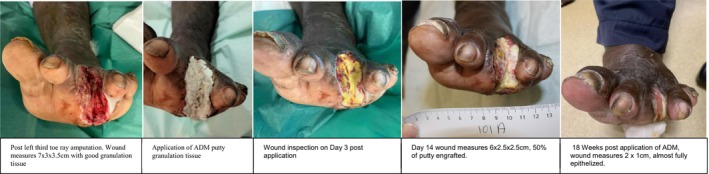
Case 2.

**FIGURE 5:**
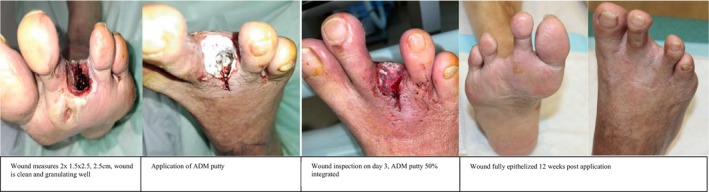
Case 3.

**FIGURE 6:**
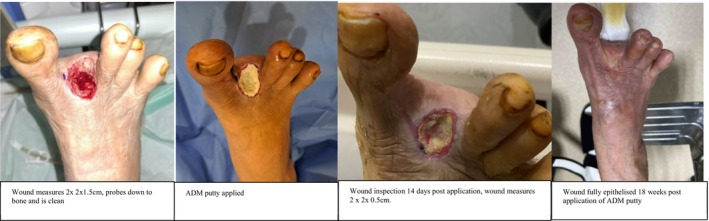
Case 4.

**FIGURE 7:**
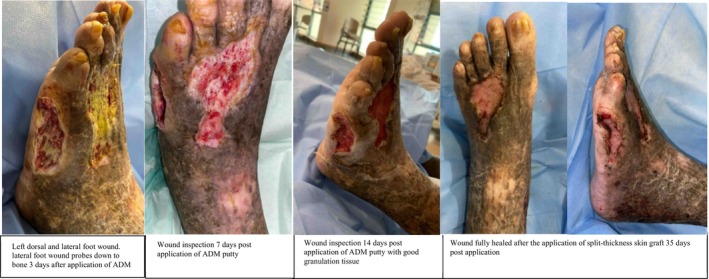
Case 5.

Patients were followed in the outpatient clinic until complete epithelialization of the wounds was achieved, with a median healing time of 16 weeks with IQR of 9. One patient required wound resurfacing with a split‐thickness skin graft. One patient had a persistent chronic wound. Another patient required readmission to hospital for infected foot wounds of the same leg and subsequently underwent transmetatarsal amputations (Figures 1, 2, 3, 4, 5, 6, 7).

Two patients succumbed to underlying ischemic heart disease 6 months and 1 year post application of ADM putty, respectively. There were no allergic or adverse reactions to the micronized ADM putty observed.

### 3.3 Case 1

84‐year‐old female with a past medical history of diabetes mellitus (HbA1c 6.5%), hypertension, hyperlipidaemia and chronic kidney disease presented with left second toe gangrene. The left Ankle Brachial Pressure Index (ABPI) was 1.11, Left Toe Brachial Pressure Index (TBPI) 0.65 Left lower limb arterial duplex studies showed stenosis in the popliteal, peroneal artery and anterior tibial artery for which she underwent angioplasty for revascularization.

She then underwent a left second toe ray amputation which resulted in a 3 × 1.5 × 1.5 cm wound with exposed metatarsal bone. Wound cultures grew methicillin‐sensitive *Staphylococcus aureus* (MSSA), and she was started on Augmentin.

She underwent a second wound debridement and subsequent application of micronized injectable ADM putty, and the wound was dressed with negative pressure dressing. She was regularly followed up in our outpatient clinic. The wound was fully epithelized by Week 8.

### 3.4 Case 2

59‐year‐old male with a past medical history of hypertension and diabetes mellitus with a HbA1c of 9.9%. He was admitted to our hospital for left foot plantar abscess involving the second and third toe with underlying osteomyelitis. Left ABPI was 1.21, TBPI 0.99.

He underwent a left foot wound debridement and third toe ray amputation. Cultures grew *Streptococcus agalactiae*, and he was started on appropriate antibiotics. His subsequent wound inspection showed a 7 × 3 × 3.5 cm wound with good granulation tissue. Acellular dermal matrix putty was applied on the wound on post‐operative Day 3, and the wound was dressed with conventional dressing.

He was reviewed regularly at outpatient clinic and underwent dressing change every alternate day due to high level of exudates. Wound was almost fully epithelized at 18 weeks.

### 3.5 Case 3

62‐year‐old male with a past medical history of diabetes mellitus with a HbA1c of 7.8%, ischaemic heart disease and peripheral vascular disease. He presented with right third toe gangrene that progressively worsened over 2 months. Right TBPI 0.29, ABPI was 2.08 and the right lower limb arterial duplex showed stenosis over the anterior tibial artery, peroneal and posterior tibial artery. He underwent a right lower limb angioplasty for revascularization and subsequent right third toe ray amputation.

Wound cultures grew *S. aureus*, and he was started on appropriate antibiotics.

Wound inspection on post‐operative Day 3 showed a 2 × 1.5 × 2.5 cm wound with good granulation tissue. ADM putty was applied and dressed with conventional dressing. He was discharged well on post followed up in our outpatient clinic. Wound fully epithelized in week 12 post application.

### 3.6 Case 4

76‐year‐old female with a past medical history of diabetes mellitus with HbA1c of 7.3, hypertension, hyperlipidaemia, stroke and previous left hip fracture and osteoporosis. She presented with a 3‐month history of worsening right toe gangrene. Right ABPI 1.57 TBPI 0.31 Arterial duplex shows occlusion of the right dorsalis pedis artery and underwent right lower limb angioplasty, right toe ray amputation. Wound cultures were positive for *Pseudomonas aeruginosa*, and she was started on antibiotics and her wound was dressed with conventional dressing. Wound inspection on post‐operative Day 7 showed a 2 × 2 × 1.5 cm wound which was clean and probed down to bone. ADM putty was applied and was dressed with conventional foam dressing. She was discharged and followed up in our outpatient clinic. The wound was full epithelized 18 weeks post application of ADM putty.

### 3.7 Case 5

73‐year‐old male with past medical history of diabetes mellitus and Hba1c of 6.4, and complete heart block with a pacemaker. He has a long‐standing history of peripheral arterial disease and chronic venous insufficiency complicated by lower limb stasis eczema and chronic mixed arteriovenous bilateral lower limb ulcers over bilateral feel for over 10 years. In recent years, this has been treated previously with left common iliac and external iliac vein stenting bilateral lower limb angioplasty and foam sclerotherapy. He presented with infected bilateral lower limb mixed arteriovenous ulcers.

His wounds include a left dorsal foot ulcer, left lateral foot ulcer exposing the fifth metatarsal head with evidence of osteomyelitis over the fifth metatarsal head, right lower limb ulcer over the dorsum and right lateral malleolus.

Arterial duplex showed stenosis over the tibioperoneal trunk, posterior tibial and anterior tibial artery. Angioplasty was performed, and the patient underwent bilateral lower limb wounds debridement and was placed on negative pressure dressings. Wound cultures were positive for morganella, proteus and enterococcus, and antibiotics was started.

On post‐operative Day 2, wounds were clean and ADM putty was applied. Wound inspection 14 days post application showed healthy granulation tissue with no critical structures exposed, and wounds were resurfaced with split‐thickness skin graft. Wounds were fully healed on subsequent wound inspection with no graft loss.

## 4 DISCUSSION

In this study, we investigate the real‐world effectiveness of an injectable human‐derived micronized putty ADM in the treatment of diabetic foot wounds. This novel ADM has the advantages of being in putty (paste) form allowing easy application in cavities. This paper is also the first reported use of the ADM outside of Korea which is the country of origin. We demonstrate statistically significant reductions in wound volume after the application of ADM in DFWs on post‐operative Days 14 and 28. Most patients were able to achieve complete eventual healing of DFW.

DFWs are challenging to treat due to their chronic nature and high recurrence rates, often leading to poor healing, infection and the risk of limb amputation. Prompt treatment and successful healing can prevent debilitating amputations. However, healing in diabetic foot wounds is arrested in the inflammatory phase due to high levels of proinflammatory cytokines, proteases and reactive oxygen species (ROS), along with cellular dysfunction^8^ in the form of defective phagocytic and chemotactic activities of granulocytes. This impaired immune response allows for infection resulting in excessive recruitment of inflammatory cells that produce ROS, damaging the extracellular matrix (ECM) and critical growth factors and receptors.^9^ Damage in ECM exacerbates cellular dysfunction resulting in premature cell senesces, apoptosis, inhibition of cell proliferation, migration and angiogenesis. High serum glucose levels stimulate the production of matrix metalloproteinase (MMP) production which causes further ECM degradation.^10^

ADMs facilitate wound healing by providing an ECM structural scaffold rich in growth factors to create a favourable microenvironment for cell proliferation, migration and angiogenesis.

While various sources of ADM exist, the optimal choice is an allogenic source that most closely replicates the structure and function of the native ECM it is intended to replace.^6^ Using human‐derived ADMs also reduces the risk of immune reaction to xenogeneic proteins.

ADMs are often available in sheet form; however, this is difficult to apply on irregular surfaces. DFUs are often irregularly shaped, with deep sinus tracks, crevices and areas of undermining. Injectable micronized ADM offers the distinct advantage of filling these deep crevices in 3 dimensions. This allows for the delivery of growth factors to hard‐to‐reach areas, promoting healing and easy application. The ease of application reduces the technical complexity, lowering the learning curve and making it more accessible for clinicians to adopt.

The micronized ADM used in this study only requires one application, making it more cost‐effective and convenient for the clinician. It is noted that the rate of wound healing in the first 14 days is twice that of the subsequent 14 days. Rate of wound healing from Days 1–14 occurred at average 3.46% per day compared with 1% per day from Days 15–28. Lee et al. also investigated the use of micronized ADM and postulated that its use recruits healing components in the early phase of wound healing,^11^ and these findings are reflected in our results. Repeat application once the product has been engrafted may further accelerate wound healing.

During our study, we also note that a significant portion of our patients had a portion of positive cultures at the onset of ADM application, although there was no overt clinical infection after meticulous debridement. Close to 70% of DFUs are often plagued by biofilm.^12^ These rates reported in literature mimic those in our study, with 87% of patients having positive wound cultures, despite the appearance of a clean granulating wound. Our study shows that ADM application and engrafting was not negatively impacted by the positive wound cultures and reduction of wound size was achieved despite this. It is well known that the ECM plays an integral part in adhesion and migration of immune cells.^13^ We postulate that the application of ADM provides a scaffold that allows for subsequent infiltration of immune cells and repair cells. The surrogate ECM also functions as a scaffold for immune cell adhesion,^14^ improving the overall microenvironment to resist bacterial proliferation and subsequent infection.

Limitations of this study include the small sample size, heterogeneity of wound types and retrospective nature of the study.

## 5 CONCLUSION

In conclusion, the application of micronized injectable human‐derived ADM is effective in promoting wound closure in DFW. Its advantages include the paste (putty) formulation allowing for application into irregular wounds. The significant reductions in wound volume support the potential of this treatment modality as an adjunct to standard wound care.

## CONFLICT OF INTEREST STATEMENT

The authors declare no conflicts of interest.

## Data Availability

The data that support the findings of this study are available from the corresponding author upon reasonable request.
